# Molecular Confirmation of *Accipiter* Birds of Prey as Definitive Hosts of Numerous *Sarcocystis* Species, including *Sarcocystis* sp., Closely Related to Pathogenic *S*. *calchasi*

**DOI:** 10.3390/pathogens12060752

**Published:** 2023-05-23

**Authors:** Tautvilė Šukytė, Dalius Butkauskas, Evelina Juozaitytė-Ngugu, Saulius Švažas, Petras Prakas

**Affiliations:** Nature Research Centre, Akademijos 2, 08412 Vilnius, Lithuania; tautvile.sukyte@gamtc.lt (T.Š.); dalius.butkauskas@gamtc.lt (D.B.); evelina.ngugu@gamtc.lt (E.J.-N.); saulius.svazas@gamtc.lt (S.Š.)

**Keywords:** *Sarcocystis*, *Sarcocystis calchasi*, *Accipiter* hawks, DNA analysis, life cycle, predator–prey, *ITS1*, phylogeny

## Abstract

The present study aimed to test intestinal scrapings of the Northern Goshawk (*Accipiter gentilis*) and the Eurasian Sparrowhawk (*Accipiter nisus*) from Lithuania for *S*. *calchasi* and other *Sarcocystis* species characterised by bird–bird life cycles. The protozoan parasite *Sarcocystis calchasi* can cause respiratory and neurological diseases in a variety of birds; however, the distribution of this parasite is not well-examined. *Sarcocystis* species were identified with nested PCR and sequencing of the partial *ITS1* region. Sporocysts and/or sporulated oocysts of *Sarcocystis* spp. were observed in 16 (100%) Northern Goshawks and 9 (56.3%) Eurasian Sparrowhawks. Four species, *S*. *columbae*, *S*. *halieti*, *S*. *turdusi*, and *S*. *wobeseri*, were confirmed in the Eurasian Sparrowhawk. Apart from the latter four species, *S*. *calchasi*, *S*. *cornixi*, *S*. *kutkienae*, and *S*. *lari* were established in the Northern Goshawk. A higher prevalence of *Sarcocystis* spp. and species richness in Northern Goshawks is associated with the differences in the diet of two examined *Accipiter* species. This study is the first report of *S*. *calchasi* in Lithuania. Furthermore, the genetically distinct species *Sarcocystis* spp. 23LTAcc, which is most closely related to *S*. *calchasi*, was found in three Northern Goshawks.

## 1. Introduction

Protozoans of the genus *Sarcocystis* (Apicomplexa: Sarcocystidae) are abundant and worldwide-distributed parasites that might infect reptiles, birds, and mammals, including man [1]. They are characterised by an obligatory two-hosts life cycle. Sarcocysts mainly develop in the muscles and CNS of the intermediate host (prey), and sporulation of oocysts occurs in the small intestine of the definitive host (predator, scavenger) [2]. The morphology of sarcocysts is the main phenotypic feature used for the differentiation of *Sarcocystis* species, whereas parasite species cannot be discriminated by the morphometric parameters of oocysts and sporocysts [1,2].

Numerous *Sarcocystis* species have been described in the muscles of birds belonging to different orders [1,3,4,5,6,7,8,9,10]. Two *Sarcocystis* species, *S*. *falcatula* and *S*. *calchasi*, are the main agents of this group of parasites that cause diseases in birds [3,4,11,12,13,14,15,16,17,18,19,20,21,22,23]. Definitive hosts of *S*. *falcatula* are opossums, and this *Sarcocystis* species is prevalent in North and South America [24,25,26]. *Sarcocystis falcatula* causes lung injuries and severe neurological symptoms in birds of numerous orders, including Accipitriformes [11,21], Charadriiformes [22], Columbiformes [3,21,27], Coraciiformes [28], Cuculiformes [21], Passeriformes [3,21,29], Pelecaniformes [13], Piciformes [21], Psittaciformes [3,21,30,31,32,33,34,35,36,37,38], Sphenisciformes [39], Strigiformes [21,40], and Suliformes [17,22]. *S*. *calchasi* was initially found in pigeons and doves and can cause neurological diseases called pigeon protozoal encephalitis (PPE) and hepatitis [4,12,15,16,17,19,20,23,41,42,43,44,45]. Infection with *S. calchasi* can result in high morbidity and mortality rates in some bird populations, particularly in captive or domesticated birds, such as racing doves or parrots in aviaries [15,16,19,20,23,41,42,43,44,45]. This pathogen was reported in Germany [4,14,16,45,46], Finland [19], Japan [47], and the USA [12,15,17,20,23,48], infecting birds of the orders Columbiformes [4,12,15,16,41,42,43,44,45,46,47,48], Galliformes [20], Piciformes [14], Psittaciformes [19,23,44], and Suliformes [17]. Recently, encephalitis associated with *S*. *halieti* has been reported in the Little Owl (*Athene noctua*) [49]. Thus, *S*. *halieti* may also be an important pathogenic species for birds.

Definitive hosts of *S. calchasi* and *S. halieti* are hawks, mainly of the genera *Accipiter* [4,5,18,46,50,51,52]. These birds of prey become infected by ingesting infected tissues (usually muscle) of various birds, which are intermediate hosts that harbour the asexual stages of the parasite [1,50,51,52]. In Lithuania, *S*. *halieti* was detected in the muscles of the Great Cormorant (*Phalacrocorax carbo*) [53], the Herring Gull (*Larus argentatus*) [54], the Hooded Crow (*Corvus cornix*), and the Common Raven (*Corvus corax*) [55]. Two species of hawks, the Northern Goshawk (*Accipiter gentilis*) and the Eurasian Sparrowhawk (*Accipiter nisus*), which may act as definitive hosts of pathogenic avian *Sarcocystis* species, are widespread and common in Lithuania [56,57]. The estimated breeding population of the Northern Goshawk (further in the text: Goshawk) is 500–800 pairs, while that of the Eurasian Sparrowhawk (further in the text: Sparrowhawk) is 2000–4000 pairs [57]. Based on geographical reports of the parasite and the distribution of intermediate and definitive hosts, it could be expected that *S. calchasi* might be prevalent in Lithuania. However, this *Sarcocystis* species has not been recorded in Lithuania yet.

The definitive hosts of *Sarcocystis* species are disclosed using laboratory experiments [58,59,60,61]. In the last two decades, molecular methods have become an important tool for gaining more knowledge of the life cycle of various *Sarcocystis* spp. Based on phylogenetic results, a group of animals (for instance, birds, mammals, and reptiles) can be suggested as possible definitive hosts of certain *Sarcocystis* species [7,8,50,51,52,53,62]. Furthermore, by means of molecular methods, *Sarcocystis* species are identified in small intestine scrapings of naturally infected predators and scavengers [50,51,52,63,64,65,66,67]. In numerous studies, it has been shown that *ITS1* (internal transcribed spacer 1) is the best marker for the identification of *Sarcocystis* species using birds as intermediate hosts [6,7,8,53,54,55,62,67].

There is a lack of knowledge about which species of *Sarcocystis* are spread by birds of prey belonging to the genus *Accipiter*. Previously, based on molecular methods, *Sarcocystis* spp. have been identified in the intestinal tract of *Accipiter* hawks in Germany [50] and the USA [18]. Therefore, the aim of the present study was to examine intestinal samples of Goshawks and Sparrowhawks collected in Lithuania for the presence of *S. calchasi* and other *Sarcocystis* species employing birds as their intermediate and definitive hosts. *Sarcocystis* species were identified using species-specific PCR and sequencing of amplified fragments.

## 2. Materials and Methods

### 2.1. Sample Collection

A total of 32 birds (16 Goshawks and 16 Sparrowhawks) were collected between 2016 and 2022. All birds were found dead (as a result of collisions with motor vehicles, power lines, buildings, etc.) and obtained from the Kaunas T. Ivanauskas Zoology Museum, the Lithuanian national authority responsible for monitoring dead birds. Birds were kept frozen at −20 °C until the animals were dissected.

### 2.2. Isolation of Oocysts/Sporocysts

*Sarcocystis* spp. were extracted from the entire intestine of each *Accipiter* bird using a modified Verma et al. [68]. technique. This technique allows isolation of different development stages of *Sarcocystis* spp., i.e., sporocysts, sporulated oocysts, and unsporulated oocysts, in definitive hosts. The small intestine was removed from the bird, cut lengthwise, and spread over the dissection table with the luminal side up. The intestinal epithelium was lightly scraped with the help of glass slides and suspended in 50 mL of distilled water (dH_2_O). The suspended scrapings in dH_2_O were homogenized in a commercial blender at top speed for 1–2 min or more, with breaks to prevent frothing. The homogenate was centrifuged for 6 min at 1600 rpm at 20 °C in 50 mL centrifuge tubes. The supernatant was discarded, sediments were re-suspended in 50 mL of water, and the homogenate was repeatedly homogenized in a commercial blender and centrifuged at the same conditions. The centrifugation and decantation were repeated until most sporulated oocysts and sporocysts were released from the host tissue. At this stage, the obtained sediments were examined for oocysts/sporocysts under a light microscope using at ×400 magnification. The 400 μL of re-suspended sediments were taken from each sample and used for DNA extraction. The isolation of DNA was performed on all bird samples, regardless of whether *Sarcocystis* spp. oocysts/sporocysts were found in them.

### 2.3. Molecular Identification and Phylogenetic Analysis

The intestinal samples of birds collected in Lithuania were tested for the presence of ten *Sarcocystis* spp., *S*. *calchasi*, *S*. *columbae*, *S*. *cornixi*, *S*. *corvusi*, *S*. *fulicae*, *S*. *halieti*, *S*. *kutkienae*, *S*. *lari*, *S*. *turdusi*, and *S*. *wobeseri*, using birds as their intermediate hosts. Birds belonging to different orders are intermediate and definitive hosts of these *Sarcocystis* species [4,5,7,50,51,52,53,62,69,70,71,72].

The purification of the genomic DNA from the sediments of intestinal scrapings was performed using the GeneJET Genomic DNA Purification Kit (Thermo Fisher Scientific Baltics, Vilnius, Lithuania) according to the manufacturer’s instructions. The DNA samples were kept frozen at −20 °C for further molecular analysis.

The identification of *Sarcocystis* species was carried out using partial sequences of the *ITS1* region, situated between the 18S rRNA and 5.8S rRNA genes. To amplify DNA fragments of the surveyed parasite species, nested PCR was used. In the first step, a forward SU1F and reverse 5.8SR primer pair was used [73]. These primers are suitable for the amplification of *Sarcocystis* spp. isolated from various hosts. In the second step of nested PCR, species-specific primers developed in our previous study were applied [65]. Additionally, for the diagnosis of *S*. *calchasi*, the newly designed primers GsScalF2 (5′-CCTTTTGTAAGGTTGGGGACATA-3′)/GsScalR2 (5′-GCCTCCCTCCCTCTTTTTG-3′) were used. The following primers were chosen using the Primers 3 Plus program [74]. A negative control (nuclease-free water instead of target DNA) and a positive control (DNA of each *Sarcocystis* spp., except of *S*. *calchasi*, extracted from single sarcocysts) were used in each round of PCR. Overall, three negative controls were used: (i) from the first amplification step (ii), from the second amplification step, and (iii) two µL of solution obtained from the negative control of the first nested-PCR step was transferred to the negative control of the second amplification step.

PCR reactions were carried out using DreamTaq PCR Master Mix (Thermo Fisher Scientific Baltics, Vilnius, Lithuania) according to the manufacturer’s instructions. The PCR cycling conditions were as follows: initial denaturation for 5 min at 95 °C; 35 cycles of 45 s at 94 °C; 45 s at 55–65 °C, depending on the primer pair; 60 s at 72 °C; and final extension for 10 min at 72 °C. All positive PCR samples were sequenced, and the DNA fragments obtained were used for the confirmation of *Sarcocystis* species. The visualization, purification, and sequencing of amplified products were carried out using a previously described protocol [8]. The obtained *ITS1* sequences of *Sarcocystis* spp. were deposited in GenBank with accession numbers OQ848675–OQ848736.

### 2.4. Analysis of DNA Sequence and Statistical Data

The obtained chromatograms were manually analysed in Chromas 2.6.5 for ambiguously placed nucleotides. The resulting sequences were truncated to exclude nucleotide binding sites. The sequences were then compared with each other and with the sequences of various *Sarcocystis* species available in GenBank by nucleotide BLAST (e.g., http://blast.ncbi.nlm.nih.gov/ (accessed on 6 April 2023)). Within-group mean genetic distances, as well as between-group mean genetic distances, were calculated using MEGA7 [75]. The Tamura 3-parameter [76] was set for the estimation of genetic distances. Phylogenetic analysis was also performed with MEGA7. Sequences were aligned with the MUSCLE algorithm [75]. The general time reversible evolutionary model [77] with gamma distribution (GTR + G) was determined to be the most suitable for the analysed alignment. For the evaluation of the robustness of the implied phylogeny, a bootstrap test with 1000 replicates was performed.

The prevalence of distinct *Sarcocystis* species established in two birds of prey, the Goshawk and Sparrowhawk, was compared using the unconditional exact test, which is a good choice when comparing small samples [78]. Statistical analysis was conducted with Quantitative Parasitology 3.0 software [79].

## 3. Results

### 3.1. Microscopical Examination of Sarcocystis spp. Oocysts/Sporocysts Isolated from Accipiter Hawks

**Figure 1:**
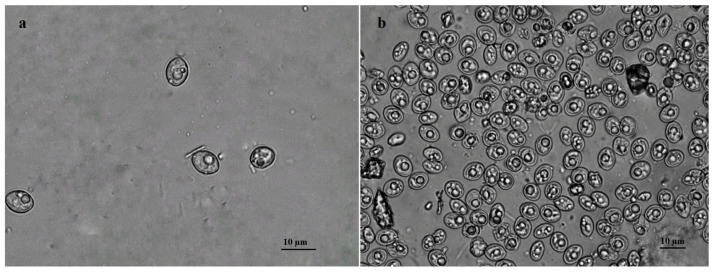
Sporulated oocysts/sporocysts of *Sarcocystis* spp. found in small intestine mucosal scrapings of Goshawk (**a**) and Sparrowhawk (**b**).

Examination of the intestinal mucosa samples of *Accipiter* hawks under a light microscope revealed *Sarcocystis* spp. infection in 25/32 (78.1%) tested birds. All examined Goshawks harboured sporocysts and/or sporulated oocysts of *Sarcocystis* spp. (16/16, 100%). Through microscopical analysis, *Sarcocystis* spp. were detected in 9 of 16 (56.3%) tested Sparrowhawks. No unsporulated oocysts were detected in both species investigated. Free sporocysts of *Sarcocystis* spp. in mucosal scrapings of Goshawk measured 13.0 × 9.2 μm (10.5–15.6 × 6.6–11.8 µm; *n* = 146) (Figure 1a), whereas sporulated oocysts were ellipsoidal in shape, contained two sporocysts, and measured 12.3 × 18.3 μm (10.5−14.5 × 11.7−21.7; *n* = 63). Meanwhile, sporocysts of *Sarcocystis* spp. in Sparrowhawk measured 11.9 × 8.1 μm (10.7–13.9 × 7.2–9.2; *n* = 130) (Figure 1b), while sporulated oocysts were 10.7 × 14.1 (10.6–13.6 × 14.0–17.0; *n* = 41) in size. Thus, the sporocysts and sporulating oocysts found in different species of hawks overlapped in size.

### 3.2. Molecular Identification and Phylogenetic Analysis of Sarcocystis spp. Closely Related to S. calchasi

Four samples isolated from Goshawks were amplified using the GsScalF/GsScalR primer pair, which was previously created to identify *S*. *calchasi* [65]. However, the comparison of the resulted sequences showed a match with *S*. *calchasi* only in one analysed isolate (AgLT7), whereas three other isolates (AgLT3, AgLT6, and AgLT9) were attributed to *Sarcocystis* spp. 23LTAcc. In the present study, designed GsScalF2/GsScalR2 primers were used to amplify a single AgLT7 isolate, and the resulted sequence (OQ848675) displayed 100% identity to sequences of *S*. *calchasi* (FJ232948) from the racing pigeon (*Columba livia domestica*).

Three 427 bp-long *ITS1* sequences of *Sarcocystis* spp. 23LTAcc (OQ848676–OQ848678) showed 100% identity between each other and the greatest similarity with *S*. *calchasi* and *S*. *wobeseri* (Table 1). It is noteworthy that the interspecific and intraspecific genetic similarity of the compared *Sarcocystis* spp. did not overlap. Additionally, the genetic differences of *Sarcocystis* spp. 23LTAcc as compared with other *Sarcocystis* spp. were more than 4% within *ITS1*. Therefore, it is very likely that *Sarcocystis* spp. 23LTAcc represent a genetically new non-described *Sarcocystis* species.

**Figure 2:**
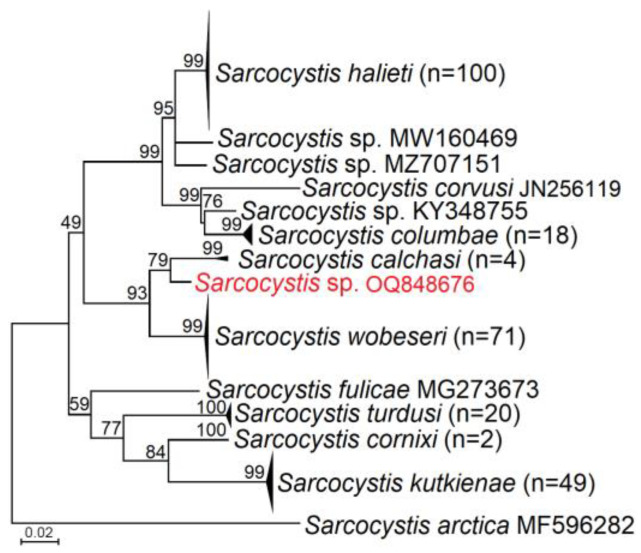
The phylogenetic tree of selected *Sarcocystis* spp. showing the close relationships of *Sarcocystis* spp. (displayed in red) obtained in the current work with *S*. *calchasi*. Since three sequences of *Sarcocystis* spp. were identical (OQ848676–OQ848678), only one was used for the phylogenetic inference. The tree was constructed based on *ITS1* sequences and using the maximum likelihood method. The bootstrap support vales are presented next to branches. The number of sequences are shown in brackets. The multiple sequence alignment contained 271 taxa and 471 aligned nucleotide positions, including gaps.

In the phylogenetic tree, *Sarcocystis* spp. 23LTAcc was placed together with a dozen *Sarcocystis* spp. employing birds as their intermediate and definitive hosts (Figure 2). The analysed *Sarcocystis* species were represented by a range from 1 to 100 sequences. Isolates of S. *halieti*, *S*. *columbae*, *S*. *calchasi*, *S*. *wobeseri*, *S*. *turdusi*, *S*. *cornixi*, and *S*. *kutkienae*, which were represented by more than one sequence, formed monophyletic clades with high bootstrap support (99–100). Based on the partial *ITS1*, *Sarcocystis* spp. 23LTAcc was a sister species to *S*. *calchasi*, and these two taxa made a sister clade to *S*. *wobeseri*. The grouping of the following three species into one cluster was supported by a high bootstrap value (93). In addition, the placement of several *Sarcocystis* spp., *S*. *halieti*, *Sarcocystis* spp. from Chilean Skua (*Stercorarius chilensis*) (GenBank: MW160469), *Sarcocystis* spp. from Common Raven (GenBank: MZ707151), *S*. *corvusi*, *Sarcocystis* spp. from Cooper’s Hawk (*Accipiter cooperi*) (GenBank: KY348755), and *S*. *columbae* into one cluster was supported by the close-to-the-maximum bootstrap value. By contrast, the grouping of the four remaining *Sarcocystis* spp., *S*. *fulicae*, *S*. *turdusi*, *S*. *cornixi*, and *S*. *kutkienae*, was not well-supported (59 bootstrap values).

### 3.3. The Identification and Distribution of Sarcocystis spp. in Intestines of Accipiter spp.

**Figure 3:**
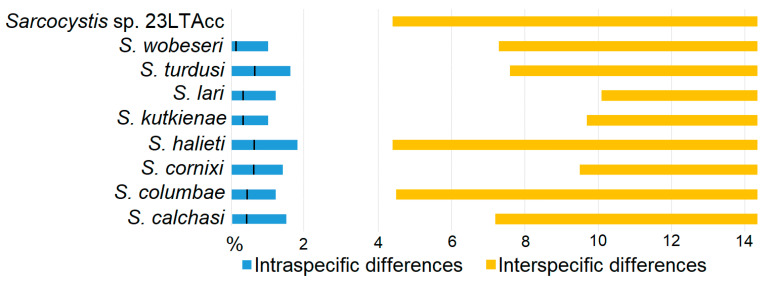
The comparison of intraspecific and interspecific genetic differences of identified *Sarcocystis* spp. within analysed fragments of *ITS1* region. The black vertical lines display the mean vales of intraspecific genetic differences expressed in percentages.

By using nested PCR targeting the *ITS1* region and subsequent Sanger sequencing, eight previously known species (*S*. *calchasi*, *S*. *columbae*, *S*. *cornixi*, *S*. *halieti*, *S*. *kutkienae*, *S*. *lari*, *S*. *turdusi*, and *S*. *wobeseri*) and *Sarcocystis* spp. 23LTAcc were confirmed in the small intestines of two *Accipiter* species (Figure 3). Interspecific genetic differences of analysed *Sarcocystis* spp. were higher than 4%. Three species, *S*. *cornixi*, *S*. *kutkienae*, and *S*. *lari*, were genetically most distant from other species within *ITS1* (possessing more than 9% interspecific genetic differences). By contrast, the lower interspecific genetic differences were calculated comparing *S*. *columbae*, *S*. *halieti*, and *Sarcocystis* spp. 23LTAcc. In general, for all examined *Sarcocystis* spp., the difference between the highest interspecific and intraspecific differences was more than 2%. Apart from *Sarcocystis* spp. 23LTAcc, other tested *Sarcocystis* spp. were characterised by up to 1.8% intraspecific genetic variation. Even so mean values of intraspecific genetic differences were small and ranged between 0.1–0.6%. Thus, in the present study, the detected species can be reliably distinguished from other *Sarcocystis* spp. with the suggested molecular method.

**Figure 4:**
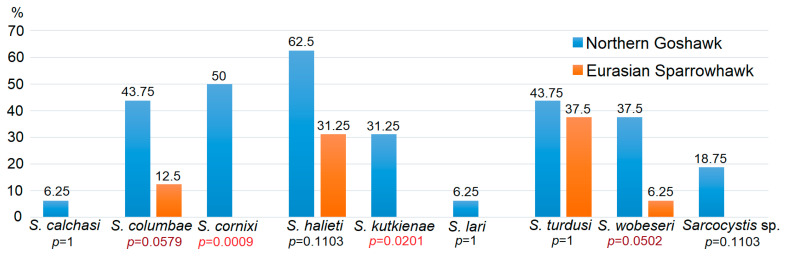
The distribution of *Sarcocystis* species in the small intestines of Goshawk and Sparrowhawk from Lithuania on the basis of the *ITS1* sequence data. Comparison of the prevalence of certain *Sarcocystis* species between two host species are presented below species names. *Sarcocystis* spp. = *Sarcocystis* spp. 23LTAcc. Statistically significant values are presented in red and marginal *p* values are shown in purple.

Overall, 48 and 14 sequences of *Sarcocystis* spp. were obtained by examining the Goshawk and Sparrowhawk, respectively. However, differences in the proportion of infected birds by means of DNA analysis were small compared to Goshawk 12/16 (75.0%) and Sparrowhawk 11/16 (68.8%). Two *Accipiter* hawks differed in the detected parasite species richness and infection rates of *Sarcocystis* spp. (Figure 4). Only four species were identified in the Sparrowhawk, as compared to nine *Sarcocystis* spp. determined in the Goshawk. The prevalence of certain species detected varied from 6.25% (1/16) to 62.5% (10/16). A higher infection rate of all identified *Sarcocystis* spp. was established in Goshawk. Nevertheless, statistically significant differences were determined only when comparing the prevalence of *S*. *cornixi* and *S*. *kutkienae* among two *Accipiter* species, while marginal differences were estimated in the case of *S*. *columbae* and *S*. *wobeseri*.

## 4. Discussion

### 4.1. Determination of the Definitive Hosts of Sarcocystis spp.

In the current study, sporulated oocysts and free sporocysts were detected in the intestinal mucosa of Goshawk and Sparrowhawk (Figure 1). The morphometric sizes of parasite sexual stages detected in *Accipiter* hawks overlapped. Thus, morphologically, it was impossible to determine how many different *Sarcocystis* species were present in the investigated samples. It is well known that birds of prey can be simultaneously infected with sporocysts of several *Sarcocystis* spp. [18,50,51]. In general, the transmission experiments reveal the definitive host of *Sarcocystis* species [1,58,59,60,61]. The need for an infection experiment raises ethical questions regarding the non-pathogenicity or low pathogenicity of the investigated *Sarcocystis* species, whereas molecular methods can be advantageous for the identification of definitive hosts of *Sarcocystis* spp. It is important to mention that the molecular identification of *Sarcocystis* species in intestinal samples does not conclusively prove that the analysed predator or scavenger is the definitive host of the parasite [51]. Furthermore, the obtained molecular analysis data on the definitive hosts of certain *Sarcocystis* species should be indirectly combined with ecological data [65,66,67].

### 4.2. Distribution of Detected Sarcocystis spp. in the Definitive Hosts

Sporulated oocysts and free sporocysts of *Sarcocystis* spp. were observed in both investigated *Accipiter* species. Based on the comparison of the obtained *ITS1* sequences, nine *Sarcocystis* species (*S*. *calchasi*, *S*. *columbae*, *S*. *cornixi*, *S*. *halieti*, *S*. *kutkienae*, *S*. *lari*, *S*. *turdusi*, *S*. *wobeseri*, and *Sarcocystis* spp. 23LTAcc) were identified in the intestinal samples of birds of prey in Lithuania for the first time (Table 2). Furthermore, this is the first confirmation of *S*. *wobeseri* and *S*. *kutkienae* in predatory birds. The prevalence of *Sarcocystis* spp. in the Goshawk obtained using morphologic methods (100%) was higher than detected by DNA analysis (75.0%). Such findings can be explained by the fact that, unlike the Sparrowhawk, the Goshawk is a generalist predator, and small mammals comprise a part of its diet. In southern Finland, small mammals (mostly squirrels, hares, and voles) formed 10% of all prey items for Goshawk [80].

Notably, the distribution of the identified *Sarcocystis* species in the current work was unequal in two *Accipiter* hawks (Figure 4). Two species, *S*. *cornixi* and *S*. *kutkienae*, were found only in the Goshawk (*p* < 0.05). Birds of the family Corvidae serve as intermediate hosts for both *Sarcocystis* species [62,69]. In Europe, corvids are among the few preferred prey groups of the Goshawk [81]. In southern Finland, corvids, mostly the Hooded Crow and the Magpie (*Pica pica*), formed more than 30% of the total diet of the Goshawk, particularly in sub-urban habitats [80]. On the contrary, Sparrowhawks do not prey on corvid birds [56].

Marginal differences (*p* = 0.0502–0.0579) were observed when comparing the higher prevalence of *S*. *columbae* and *S*. *wobeseri* in Goshawk than in Sparrowhawk. Previously, *S*. *columbae* was detected in the muscles of bird families Columbidae and Laridae [5,54,82], while up to date, anatids, larids, and accipitrids are known intermediate hosts of *S*. *wobeseri* [54,70,83]. Columbids (pigeons and doves) comprise the bulk of the Goshawk diet during the breeding season [84]. A rapid increase in Feral Pigeons (*Columba livia domestica*) was recently recorded as prey for Goshawk in urban and sub-urban habitats of Europe [80]. Columbids are the preferred prey of the Sparrowhawk [85]. In the United Kingdom, the Wood Pigeon (*Columba palumbus*), Eurasian Collared Dove (*Streptopelia decaocto*), and Feral Pigeon were identified as the key diet items of the species [86]. Larids and waterfowl species comprise a small part of the diet of tested *Accipiter* hawks [56,84]. In southern Finland, waterfowl and shorebird species formed about 3% of the total diet of the Goshawk [80].

Goshawks breeding in the eastern Baltic region are partly migratory, with most ringed individuals recorded in winter being up to 400 km from their nesting sites [56]. Birds breeding in Latvia, Estonia, and Finland were found in Lithuania in October–April. During the migration period, domestic poultry (mostly hens) form a small part of the diet of the Goshawk, while in winter, the diet includes hares and the Grey Partridge (*Perdix perdix*) [56,80]. Sparrowhawks breeding in Lithuania and in adjacent countries are short-distance migrants [56]. The diet of migratory individuals in Lithuania includes not only typical forest birds but also species of agricultural and sub-urban habitats (Starlings (*Sturnus sturnus*), House Sparrows (*Passer domesticus*), etc.) [56].

**Table 2:** Intermediate hosts and definitive hosts of *Sarcocystis* species identified in the present study (indicated by the bird family).

*Sarcocystis* spp.	Intermediate Hosts	Definitive Hosts Defined by Molecular Methods
*S*. *calchasi*	Cacatuidae [44]; Columbidae [4,12,15,16,41,42,43,44,45,46,47,48]; Numididae [20]; Phalacrocoracidae [17]; Picidae [14]; Psittacidae [19]; Psittaculidae [23]	Accipitridae [4,18,46, PS]
*S*. *columbae*	Columbidae [5,82]; Laridae [54]	Accipitridae [5,18, PS]; Corvidae [65]
*S*. *cornixi*	Corvidae [55,69]	Accipitridae [51, PS]; Corvidae [65]
*S*. *halieti*	Accipitridae [87]; Corvidae [69]; Laridae [54]; Phalacrocoracidae [53]; Procellariidae [22]; Strigidae [49]; Sturnidae [52]	Accipitridae [18,50,51,52, PS]; Corvidae [65]
*S*. *kutkienae*	Corvidae [55,62]	Accipitridae [PS]; Corvidae [65]
*S*. *lari*	Laridae [7,54]	Accipitridae [51, PS]; Corvidae [65]
*S*. *turdusi*	Turdidae [71]; Muscicapidae *	Accipitridae [18,50, PS]; Corvidae [65]
*S*. *wobeseri*	Accipitridae [83]; Anatidae [70]; Laridae [54]	Accipitridae [PS]; Corvidae [65]
*Sarcocystis* spp.	Not determined	Accipitridae [PS]

PS-present study; * see GenBank, accession numbers KJ540167 and KT588518.

In this work, *S*. *halieti* and *S*. *turdusi* were the most commonly identified species in two *Accipiter* species. Notably, *S*. *halieti* is multi-host adapted, employing birds of several different orders as intermediate hosts [22,49,51,52,53,54,55,87], while *S*. *turdusi* was found in the muscles of small passeriform birds of the families Turdidae [71] and Muscicapidae. Thrushes are the preferred prey items for the Sparrowhawk [85]. In Poland, the Song Thrush (*Turdus philomelos*) comprised 29% of the total diet of the Sparrowhawk [88]. Remarkably, as in this work, *S*. *fulicae* and *S*. *corvusi* were not found in the small intestine samples of corvids. It is assumed that *S*. *corvusi* is rare in the area under examination [55]. Whereas, in Lithuania, other birds of prey specialized in the predation of waterbirds, such as the Western Marsh Harrier (*Circus aeruginosus*) and the White-tailed Eagle (*Haliaetus albicilla*), prey on the Eurasian Coot (*Fulica atra*), which acts as an intermediate host for *S. fulicae* [56,57].

In conclusion, the composition and distribution of identified *Sarcocystis* species in the analysed birds of prey are consistent with the diet of the Goshawk and the Sparrowhawk.

### 4.3. Sarcocystis spp. Closely Related to S. calchasi

Based on DNA analysis, *Sarcocystis* spp. 23LtAcc was identified in the small intestine of three Goshawk individuals. Three identical 427 bp-long *ITS1* sequences of *Sarcocystis* spp. 23LtAcc showed 95.34–95.57% and 93.26–93.95% sequence similarity with *S*. *calchasi* and *S*. *wobeseri*, respectively (Table 1). Among the analysed avian *Sarcocystis* species, up to 1.8% intraspecific genetic variation was detected (Figure 3). Therefore, the current study indicates strong evidence that *Sarcocystis* spp. 23LtAcc represents a separate *Sarcocystis* species. Phylogenetically, *Sarcocystis* spp. 23LtAcc was most closely related to *S*. *calchasi* and, together with this species, formed a sister clade to *S*. *wobeseri*. Thus, in further studies, the nucleotide composition of *Sarcocystis* spp. 23LtAcc should be considered when identifying *S*. *calchasi*. Taking into account that *S*. *calchasi* is highly pathogenic [12,15,16,17,19,20,23,41,42,43,44,45,46,47,48], further investigations are needed to morphologically and genetically characterise *Sarcocystis* spp. 23LtAcc. Several genes, including nuclear 18S rRNA, 28S rRNA, mitochondrial cytochrome c oxidase subunit I (*cox1*), and apicoplast RNA polymerase beta subunit (*rpoB*), were mostly used for the characterisation of *Sarcocystis* species, whose intermediate hosts are birds [49,83,87]; whereas light microscopy and electron microscopy of sarcocysts are the main tools for the morphological description of *Sarcocystis* species [1,4,5,6,7,8,9,10,53,62].

Moreover, it should be clarified whether *S*. *wobeseri*, which is closely related to *S*. *calchasi*, might be pathogenic for intermediate hosts [83]. This species was detected in the leg muscles of anseriforms [70] and Herring Gulls [54] and in the pectoral and cardiac muscles of the White-tailed Eagle [83]. However, a comprehensive histopathological examination of *S*. *wobeseri* is lacking [54,83].

### 4.4. Identification of S. calchasi in Lithuania

*Sarcocystis calchasi* infection can lead to various clinical outcomes, depending on the bird species and the severity of the infection [4,12,15,16,17,19,20,23,41,42,43,44,45,46,47,48]. In pigeons, *S*. *calchasi* infection can cause neurological symptoms such as ataxia, head tilt, and torticollis [47], as well as severe diffuse, fibrinous perihepatitis [42]. In psittacines, the infection can cause neurological signs such as seizures and paralysis [89]. In Brandt’s Cormorants (*Phalacrocorax penicillatus*), the clinical signs of *S*. *calchasi* infection are not well-characterised, but it is known that the parasite can cause encephalitis and lead to neurological dysfunction in these birds [17]. The pathogenicity of *S*. *calchasi* for *Accipiter* hawks is not fully understood, but recent studies suggest that these birds of prey are a natural reservoir for this parasite species [50,51,52]. Epidemiological studies have shown that the prevalence of *S*. *calchasi* in wild bird populations can vary depending on the region and the bird species. A country-wide study in Germany demonstrated that the prevalence of *S*. *calchasi* in free-ranging *Accipiter* hawks was 7.3% (95% CI = 4.9–10.5%), with a significantly higher infection rate in juveniles (13.7%; CI = 7.7–22.0%) than in adult birds (5.8%; CI = 2.7–9.3%). Additionally, a slightly higher prevalence of *S*. *calchasi* infection was established in Goshawk (10.1%; 95% CI = 5.3–17.0%) than in Sparrowhawk (6.0%; 95% CI = 3.4–9.7%). However, the difference between these two species was not statistically significant (*p* = 0.17) [46]. In the present research, the *S*. *calchasi* species was discovered for the first time in Lithuania. However, the infection was found to be low, as only one Goshawk was confirmed to be positive for this pathogenic *Sarcocystis* species (Figure 4). Therefore, it can be assumed that *S*. *calchasi* is not widespread in Lithuania.

## 5. Conclusions

Using species-specific nested PCR targeting the partial *ITS1* region and Sanger sequencing, eight known *Sarcocystis* species, *S*. *calchasi*, *S*. *columbae*, *S*. *cornixi*, *S*. *halieti*, *S*. *kutkienae*, *S*. *lari*, *S*. *turdusi* and *S*. *wobeseri*, were identified in the small intestines of Goshawk and Sparrowhawk from Lithuania. Higher *Sarcocystis* species richness and detection rates of examined parasites were established in the Goshawk. The distribution of the detected *Sarcocystis* species was related to the diet of two *Accipiter* hawk species.

The study represents the first evidence that *Accipiter* hawks play a significant role in the distribution of *S*. *wobeseri* and *S*. *kutkienae*, producing sarcocysts in the muscles of the birds of the family Corvidae. In addition, our research provides the first report of pathogenic *S*. *calchasi* in Lithuania. Furthermore, a genetically new species, *Sarcocystis* spp. 23LTAcc, closely related to *S*. *calchasi*, was detected in three Goshawks.

## Figures and Tables

**Table 1:** The genetic comparison of *Sarcocystis* spp. 23LTAcc with *S*. *calchasi* and *S*. *wobeseri* on the basis of *ITS1* fragment. Values in the diagonal show percentage intraspecific genetic identity and mean intraspecific genetic distance in parenthesis. Vales of interspecific genetic similarity and interspecific mean genetic distances are demonstrated below and above diagonal, respectively.

Species	*Sarcocystis* spp. 23LTAcc	*S*. *calchasi*	*S*. *wobeseri*
*Sarcocystis* spp. 23LTAcc	100% (0)	0.044	0.054
*S*. *calchasi*	95.34–95.57%	98.14–100% (0.005)	0.071
*S*. *wobeseri*	93.26–93.95%	91.86–92.79%	99.07–100% (0.001)

## Data Availability

The *ITS1* sequences of *Sarcocystis* spp. from intestinal mucosa scrapings of Goshawk and Sparrowhawk were submitted in GenBank database with accession numbers OQ848675–OQ848736.
